# Response to “Are There Non-Responders to the Ergogenic 3 Effects of Caffeine Ingestion on Exercise Performance?”

**DOI:** 10.3390/nu10111752

**Published:** 2018-11-13

**Authors:** Kyle Southward, Kay Rutherfurd-Markwick, Claire Badenhorst, Ajmol Ali

**Affiliations:** 1 School of Sport, Exercise and Nutrition, Massey University, North Shore Mail Centre, Private Bag 102 904, Auckland 0745, New Zealand; K.A.Southward@massey.ac.nz (K.S.); C.Badenhorst@massey.ac.nz (C.B.); 2 School of Health Sciences, Massey University, Auckland 0745, New Zealand; K.J.Rutherfurd@massey.ac.nz; 3 Centre for Metabolic Health Research, Massey University, Auckland 0745, New Zealand

In response to “Letter: are there non-responders to the ergogenic effects of caffeine ingestion on exercise performance” by Grgic [1], we welcome the additional context that this letter provides to our paper [2]. We agree with the sentiment that responders and non-responders are misleading to readers and thus avoided using these terms in our publication [2] as much as possible. As stated by Grgic [1], an individual may perform well in one test and not another following caffeine ingestion, likewise the individual may perform better or worse on different days given the same caffeine supplementation due to multiple external factors (as mentioned in our paper [2]) and variation in performance.

With regards to the study design of future research, while it may be beneficial to use multiple exercise modes to determine the ergogenicity of caffeine, it is quite often not realistic to do so within the same study. Most studies investigating the ergogenic benefits of supplements use a specific exercise modality to answer a specific research question, for example exploring the effects of caffeine intake on endurance time-trial performance [3,4,5,6]. Including multiple exercise modalities within the same study would greatly increase the participant burden, financial costs and time to carry out the study. However, we agree that researchers should still be encouraged to use a variety of valid exercise modalities to gain a comprehensive understanding of a particular supplement. The recommendations put forward by Grgic [1] are welcomed and should be applied where applicable, particularly the reporting of individual data in response to caffeine supplementation as well as when drawing conclusions from the results.
